# Novel LHRH-receptor-targeted cytolytic peptide, EP-100: first-in-human phase I study in patients with advanced LHRH-receptor-expressing solid tumors

**DOI:** 10.1007/s00280-014-2424-x

**Published:** 2014-03-08

**Authors:** Kelly K. Curtis, John Sarantopoulos, Donald W. Northfelt, Glen J. Weiss, Kerry M. Barnhart, John K. Whisnant, Carola Leuschner, Hector Alila, Mitesh J. Borad, Ramesh K. Ramanathan

**Affiliations:** 1 Division of Hematology/Oncology, Mayo Clinic Cancer Center, 13400 East Shea Boulevard, Scottsdale, AZ 85259 USA; 2 Institute for Drug Development, Cancer Therapy and Research Center, University of Texas Health Science Center San Antonio, San Antonio, TX USA; 3 Virginia G. Piper Cancer Center, Scottsdale Healthcare (VGPCC)/TGen, Scottsdale, AZ USA; 4 Esperance Pharmaceuticals, Inc., Baton Rouge, LA USA

**Keywords:** LHRH receptor, EP-100, Cytolytic peptide, Cytolytic peptide conjugate, Advanced/metastatic solid tumors

## Abstract

**Purpose:**

To conduct a phase I study determining the safety, pharmacokinetics and preliminary efficacy of EP-100, a novel anticancer drug consisting of natural luteinizing-hormone-releasing hormone (LHRH) ligand linked to a cationic membrane-disrupting peptide.

**Methods:**

Patients with advanced, solid tumors, positive for LHRH receptor by immunohistochemistry (IHC), received EP-100 weekly or twice weekly for 3 of 4 weeks in a 28 day cycle. A modified Fibonacci 3 + 3 dose-escalation schema was used. Initial cohorts received EP-100 once weekly (cohorts 1–7, 0.6–7.8 mg/m^2^, *n* = 21). Later cohorts received doses twice weekly (cohorts 7–11, 7.8–40 mg/m^2^, *n* = 16).

**Results:**

LHRH-receptor expression was confirmed by IHC in 52 of 89 consented patients; 37 patients received at least 1 dose. Cohorts receiving doses of 5.2 mg/m^2^ and above achieved therapeutic levels from in vitro studies Clearance was rapid (mean half-life 7.1 ± 3.8 to 15.9 ± 3.6 min). The maximum-tolerated dose was not reached at the highest dose evaluated (40 mg/m^2^ twice weekly). Grade 2 increase in alanine aminotransferase/serum aspartate aminotransferase in one patient resolved, did not recur upon re-treatment, and was not observed in other patients. The only drug-related adverse event was transient infusion-related dermatologic reactions (10 patients). No complete or partial tumor responses were observed; seven patients had stable disease of 16 weeks.

**Conclusions:**

EP-100 was well tolerated in patients with advanced, LHRH-receptor-expressing solid tumors. The recommended phase 2 dose is 40 mg/m^2^ twice weekly for 3 of 4 weeks per cycle.

**Electronic supplementary material:**

The online version of this article (doi:10.1007/s00280-014-2424-x) contains supplementary material, which is available to authorized users.

## Introduction

Human cancer may be targeted via expression of luteinizing-hormone-releasing hormone (LHRH) receptors [1–18]. High LHRH receptor expression is found in diverse tumor types, including: prostate [7], breast [2, 8, 9, 12], ovarian [10, 11], endometrial [1, 8, 11], pancreatic [12], bladder [13], colorectal [14], melanoma [15], renal cancers of clear cell and chromophilic papillary histology [16] and non-Hodgkin lymphoma [18]. Malignant cells typically display LHRH-receptor overexpression relative to their normal counterparts, except anterior pituitary basophil cells. LHRH receptor typically is not expressed by vital organs [19]. LHRH conjugates are being developed to deliver cytotoxic molecules selectively to malignant cells expressing LHRH receptors. Examples include LHRH conjugates with bovine RNase A [20], pokeweed protein [21, 22] and *Pseudomonas* exotoxin [23]. Proteins involved in apoptosis (e.g., BIK, BAK, BAX and DFF40) have been conjugated to LHRH [24]. Conjugates of LHRH with cytotoxic agents such as doxorubicin, paclitaxel, melphalan, cisplatin and methotrexate are under active investigation [25, 26].

One disadvantage of this approach is the release of toxins conjugated to LHRH into systemic circulation, possibly leading to toxicity. Lytic peptides conjugated to LHRH act rapidly, independently of drug resistance and target-cell proliferation, do not require release of the toxic domain by linker cleavage, and delivery to non-LHRH-receptor-overexpressing cell membranes is limited, reducing the natural antigenicity and hemolytic activity of lytic peptides.

Lytic peptides contain an α-helical domain that interacts with negatively charged membranes, causing cell death through membrane lysis [27–29]. Internalization and mitochondrial toxicity may also be involved [30]. Plasma membrane disruption [31] and apoptosis via upregulation of death effectors [32] or by mitochondrial membrane permeabilization or change of mitochondrial membrane potential [28, 33] are proposed mechanisms by which lytic peptides cause cell death.

EP-100 is a synthetic 28 amino acid consisting of the LHRH natural ligand joined to an 18 amino acid cationic α-helical lytic peptide (CLIP-71) without a linker. LHRH delivers the lytic peptide to cancer cells via specific binding to cell-surface LHRH receptors. Although EP-100’s exact mechanism of action has not yet been resolved, cancer cell membrane surface characteristics are believed to play a critical role. Cancer cells are highly negatively charged, containing up to sevenfold higher phosphatidyl serine (PS) levels in their outer membrane, whereas normal cells have a neutral outer membrane and contain PS only in the inner plasma membrane [34, 35], resulting in greater sensitivity of a cancer cell membrane to a cationic lytic peptide. Upon accumulation on the cell membrane via LHRH receptor targeting, it is believed that EP-100 interacts with the negatively charged membrane, causing it to disintegrate, resulting in lysis and cell death. This cytotoxic activity has been demonstrated in vitro, with cell-death proteases released from cells within minutes at low micromolar concentrations of EP-100, but not in the presence of the untargeted lytic peptide [36]. Cancer cells exposed to EP-100 showed membrane disintegration as early as 5 min, and complete cell lysis occurred after incubation with the drug for 1 h.

Pre-clinical studies of EP-100 have demonstrated in vitro activity at sub-micromolar levels in various human cancer cell lines from solid tumors and hematological malignancies expressing the LHRH receptor, [Esperance data on file, 37]. In vivo activity was demonstrated in mice implanted with LHRH-receptor-positive human ovarian cancer cell line (OVCAR-3), where three weekly doses given as single bolus injection as low as 0.2 mg/kg resulted in decreased CA-125 levels and tumor regression. Unconjugated lytic peptide was inactive [36], indicating that a high density of LHRH receptors is necessary for sufficient delivery of lytic peptide to cause cell lysis (necrosis). Repeated dose toxicology studies showed that doses as high as 2.0 mg/kg were well tolerated in mice and dogs (Esperance data on file). The studies demonstrated expected pharmacologic effects of EP-100, including a reduction in pituitary production of luteinizing hormone (LH) and follicle-stimulating hormone (FSH) in some animals, which was associated with reduction in size of reproductive tissues (uterine and epididymal weights) in females and males.

The EP-100 profile of safety, pharmacokinetics (PK), pharmacodynamics (PD) and antitumor activity provides the basis for therapeutic human trials. Here, we report the first-in-human study of EP-100 in subjects with advanced solid tumors expressing LHRH receptor.

## Methods

### Study design and dose escalation

An open-label dose-escalation study was conducted at three centers after review and approval by each centers’ Institutional Review Board. Patients’ written informed consent was provided prior to study enrollment. The primary objectives of the study were to determine the maximum-tolerated dose (MTD) of EP-100 by intravenous (IV) infusion and to describe any dose limiting toxicities (DLT), which would inform the recommended phase 2 dose (RP2D) of EP-100 for future phase 2 protocols. Secondary objectives included: (a) to profile the PK and PD of EP-100, (b) to assess any drug immunogenicity by antidrug antibodies (ADA) which may react with endogenous LHRH, (c) to monitor biomarkers of pharmacologic activity of EP-100 and (d) to assess antitumor activity of EP-100 by radiographic assessments.

Cohorts of three patients were enrolled in a 3 + 3 dose-escalation design. The highest non-severely toxic dose from repeat-dose animal studies was 2.0 mg/kg/dose; BSA conversion to human mg/m^2^ suggested 40 mg/m^2^, and translation by a FDA safety factor for cytotoxic agents [38] gave a safe human by starting dose of 6.6 mg/m^2^ or 0.17 mg/kg for a 65-kg patient. A conservative first-in-human starting dose of 0.6 mg/m^2^ was chosen, based on the repeat-dose toxicity studies in dogs (which appeared to be the most sensitive species). The initial dose schedule of one 30 min infusion weekly was predicted from animal PK profiles. Dosing frequency and infusion time were increased in subsequent cohorts by two protocol amendments. To increase the cumulative dose-exposure to EP-100, infusions were increased to twice weekly after conducting safety assessments in the first seven cohorts (*n* = 21 patients, at dose levels 0.6–7.8 mg/m^2^). Twice weekly infusions effectively doubled the EP-100 delivered in all patients after cohort 7. The infusion time for the twice weekly doses also was increased from 30 to 60 min beginning with one patient in cohort 8 (dose level 11.7 mg/m^2^), which decreased the intensity of infusion reactions (described below).

The absence of grade 2 drug-related toxicities in lower dose cohorts allowed 100 % dose acceleration. In the event of grade 2 or higher non-DLT in one or more patients, dose was escalated by 50 %. Dose escalation was limited to 50 % for any grade 1 or 2 drug-related dermatologic reactions unlikely to be allergic reaction or histamine-release syndromes and to 25 % for any grade 3 or 4 drug-related infusion reaction. With occurrence of the first DLT, the cohort was expanded to three additional patients. If one or more of the three additional patients at that dose level experienced DLT (total of two or more patients with DLT), MTD was reached. MTD was defined as the dose level below which two out of three to six patients experienced a DLT during the first cycle or during 1 complete cycle (28 days).

Dose reduction to that previously demonstrated safe (i.e., the previous cohort) was permitted to manage a patient’s hematologic or non-hematologic toxicity. EP-100 administration was to be discontinued in case of grade 3 or 4 non-hematologic drug-related toxicity or for DLT, causing a treatment delay of more than 3 weeks. EP-100 dosing continued until disease progression, unacceptable toxicity or voluntary withdrawal from the study. Concomitant therapy with LHRH agonists was permitted.

### Patient selection

Patients were 18 years or older with histologically confirmed, LHRH-receptor-positive solid tumors. LHRH-receptor expression was confirmed by immunohistochemistry (IHC) on archival tumor tissue sent to a reference laboratory prior to study enrollment. Since pre-clinical studies showed the highest EP-100 activity in breast, ovarian, endometrial and prostate cancers and non-Hodgkin lymphoma, only patients with these cancers were selected beginning at dose level 5.2 mg/m^2^, possibly increasing the chance of clinical response at plasma concentrations efficacious in pre-clinical models.

Patients were eligible if they demonstrated LHRH-receptor-positive tumors, had a history of disease progression after standard/approved therapy and/or had no standard therapy available. The patients may not have been treated with radiation or investigational therapies within 4 weeks prior to Day 1 and should not have received chemotherapy within 3–5 half-lives of the chemotherapy agent or 4 weeks prior to Day 1. The patients believed to benefit from hormonal manipulation therapies (e.g., estrogen/progesterone receptor-overexpressing breast cancer not previously treated with hormonal therapy or prostate cancer not previously treated with anti-androgen therapy) were excluded. The patients with conditions predisposing to histamine release (e.g., mastocytosis, asthma and other allergic disorders with increased mast cell numbers) also were excluded. See Supplementary Information for full inclusion/exclusion criteria.

### Assessments

A history and physical examination were conducted pre-study and before every cycle. A complete blood count, serum electrolytes and chemistries were evaluated pre-study and then weekly. Tumor response or progression was assessed by imaging studies at baseline and after every 2 cycles. RECIST version 1.1 criteria were used to assess response.

FSH and LH levels as well as testosterone in men and estradiol levels in women were obtained on Days 1, 8, 15 and 22 within 24 h prior to dosing. Testosterone and estradiol levels were measured on Day 1 only after Cycle 1 and at off study assessment. Blood was collected for circulating tumor cells (CTC) prior to dosing on Day 1 of the first cycle. Patients with positive CTC at baseline also had blood collected for CTC analysis prior to dosing on Day 1 of each subsequent cycle. Additional studies included plasma cortisol, adrenocorticotropin (ACTH), thyroxine (T4) and thyroid-stimulating hormone (TSH) and blood for tumor specific biomarker analysis (at investigator’s discretion), all obtained prior to Day 1 dosing with each cycle and at off study assessment. Samples for antidrug antibodies (ADA) were collected on Day 1 of Cycle 1 and then on Day 1 of every odd cycle.

### Tumor LHRH-receptor expression

Immunoperoxidase staining was performed on slides from archival paraffin-embedded tissue. Tumor specimens were sent from clinical sites to a central pathology laboratory (Pathology Group of Louisiana, Baton Rouge, LA). Testing was performed using the GnRH (LHRH)-receptor mouse monoclonal antibody (Clone A9E4, Vector Laboratories, VP-G811, Burlingame, CA), and NIEW DAB Detection Kit (Ventana Medical Systems, Inc., Tucson, AZ). Immunoperoxidase stains were performed on one of the Ventana XT, Ventana Benchmark or Ventana Nexus units and analyzed utilizing the Ventana Image Analysis System, an adjunctive computer-assisted image analysis system functionally connected to an interactive microscope (Axio Imager, Carl Zeiss, NY). Quantitative analysis was performed according to the program for the HER2/neu receptor, which includes morphometric and colorimetric analysis. Receptor staining intensity was reported as 0, 1 + (1–25 %), 2 + (26–50 %) or 3 + (51–75 %), and was confirmed manually by a single experienced pathologist.

### Safety

All adverse events (AE) were graded and reported according to National Cancer Institute Common Terminology Criteria for Adverse Events version 3.0 (NCI CTCAE 3.0). DLT were recorded during Cycle 1 if patients had any of the following: grade 4 neutropenia lasting 5 or more days, grade 3/4 neutropenia with fever and/or infection, grade 4 thrombocytopenia or grade 3 thrombocytopenia with bleeding, grade 3 or 4 allergic reaction, grade 3 or 4 drug-related non-hematologic toxicity (not including grade 3 nausea/vomiting or diarrhea with sub-optimal prophylactic and curative treatment) or a dose delay of more than 2 weeks due to treatment-related AE or laboratory abnormalities. Any grade 3 AE not judged to be clinically significant (e.g., alopecia or drug-related fever) were not considered DLT.

### Pharmacokinetics and pharmacodynamics

Blood samples (approximately 5 mL) were collected on Cycle 1 Day 1 at the following times: pre-dose, 10 and 20 min after start of EP-100 infusion, prior to completion of the infusion (−3 min) and 2, 15, 30, 60 (±2), 120 (±2) and 240 (±5) min after the infusion. Concentrations of EP-100 in EDTA-anticoagulated plasma were assayed by a validated HPLC-Mass Spectrometry method developed at Covance (Indianapolis, IN). EP-100 and the internal standard were extracted from human plasma by solid-phase extraction and analyzed using liquid chromatography with tandem mass spectrometric detection (MS/MS). The lower limit of quantitation for EP-100 in human plasma was 0.025 μg/mL, with linearity demonstrable to 5 μg/mL (upper limit of quantitation). Plasma PK analysis for EP-100 was performed using non-compartmental methods with Phoenix™ WinNonlin^®^ version 6.1 or higher (Pharsight Corp., Mountain View, CA). Concentration–time profiles were constructed from the plasma sample data. Estimates of the area under the plasma concentration time curve (AUC) and slope of the terminal decay phase were used to calculate values of the following pharmacokinetic parameters: apparent terminal phase half-life (*t*_1/2_), total body clearance (CL), apparent time of maximum concentration (*T*_max_) and apparent volume of distribution (*V*_z_). Means and standard deviations are presented where applicable. Phoenix™ WinNonlin^®^ was used to identify the terminal linear phase of the concentration–time profile. The number of points chosen was determined based on the largest regression coefficient, *R* ^2^, obtained. A minimum of three data points was used for determination of *λ*_z_, the elimination rate constant, determined by the slope of the terminal linear phase. The *R* ^2^ had to be ≥0.8 to be included in the pharmacokinetic results. Summary statistics were prepared using Microsoft Excel 2007, and graphics were prepared with SigmaPlot™ version 11.2 (Systat Software Inc., Richmond, CA).

### Antidrug antibodies

ADA were evaluated using an enzyme linked immunoassay validated for the detection of antibodies against EP-100 (anti-EP-100) (Icon plc, formerly Prevalere Life Sciences, LLC, Whitesboro, NY). Antibodies were produced in rabbits against the lytic peptide moiety of EP-100 (APC 338983). Antibodies against EP-100 in human serum were detected by coating 96-well microtiter plates with peptide 338983. At least two wells from each plate were coated with human IgG at 500 ng/mL as controls for the goat antihuman horseradish peroxidase detector. Rabbit anti-EP-100 polyclonal antibody was used as a positive control. The plates were washed, and a mixture of goat antirabbit HRP and goat antihuman HRP and tetramethylbenzidine peroxide substrate were added, and the color development determined in a spectrophotometer at 450 and 620 nm wavelength. The developed color was proportional to the amount of anti-EP-100 antibody in the sample. The anti-EP-100 antibodies in serum were quantified from a calibration curve prepared from the positive control and ranged from 500 to 32,000 ng/mL in 100 % human serum.

### Statistical analyses

PK, PD, safety and tumor response data were summarized using descriptive statistics. Analysis of variance (ANOVA) was used to compare PK variables, laboratory safety results and antitumor activity at different doses and to identify trends in peak plasma drug and metabolite concentrations suggestive of drug accumulation or alterations in PK behavior.

## Results

### Patient characteristics

**Fig. 1:**
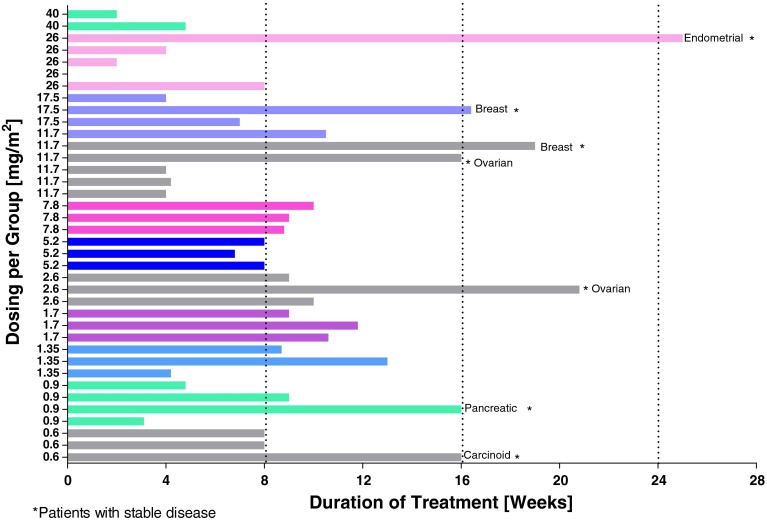
Duration of EP-100 treatment in weeks by patient at each tested EP-100 dose level. Tumor histologies of patients treated for at least 16 weeks (or beyond) are indicated

Eighty-nine (89) patients with advanced cancers were consented and had tissue blocks or slides available for LHRH-receptor screening. LHRH receptors were expressed on tumors from 52 patients (58 %). LHRH-receptor-positive expression rate was highest for breast (89 %, 17 of 19) and ovarian (65 %, 11 of 17) cancers, all of which were scored as 1+. One of two pancreatic cancers and one of four colorectal cancers scored 2+. One non-Hodgkin lymphoma and one carcinoid tumor scored 1+ for LHRH receptors. Thirty-seven patients received at least 1 dose of EP-100 as single agent. Demography for these patients is presented in Table 1. Median treatment duration was 8.7 weeks (range 2–25 weeks), with 35 patients completing at least 4 weeks. See Fig. 1 for the range of treatment duration by dose level for the 11 cohorts.

**Table 1:** Patient characteristics and demographics

	Number of patients *N* = 37		
	Female (*N* = 28)	Male (*N* = 9)	All patients
Age (years)			
Median (range)	59 (39–80)	64 (47–73)	61 (39–80)
Race			
Caucasian/white	24	9	33
American Indian or Alaska Native	2	0	2
Native Hawaiian or Pacific Islander	1	0	1
Asian	1	0	1
Performance status at baseline (Karnofsky)	90 (80–100)	90 (80–100)	90 (80–100)
Time since diagnosis of metastatic cancer to consent (months)			
Median (range)	36.5 (1–192)	18 (1–29)	27 (1–192)
Time since primary diagnosis to consent (months)			
Median (range)	71.5 (1–251)	25 (13–97)	60 (1–252)
Histology			
Breast	15		15
Ovarian, fallopian and granulosa	8		8
Endometrial	3		3
Pancreatic		2	2
Prostate		1	1
Non-Hodgkin lymphoma	1		1
Carcinoid		2	2
Colon	1	3	4
Cholangiocarcinoma		1	1
Prior treatments			
Platinum	11	5	16
Taxane	18	2	20
Platinum and taxane	11	0	11
Anthracycline	20	1	21
Antimetabolites	23	10	33
Immunotherapies	11	3	14
Anti-angiogenic agents	8	3	11
Hormone therapy	15	3	18
Investigational therapy	4	3	7
Treatment intend			
First line	1	4	5
Adjuvant	12	2	14
Neoadjuvant	7	1	8
Palliative	18	3	21

### Dose escalation

Eleven dose levels were explored: 0.6, 0.9, 1.35, 1.7, 2.6, 5.2, 7.8, 11.7, 17.5, 26 and 40 mg/m^2^. To increase drug exposure, twice weekly infusions (30 min) were given to each cohort after dose level 7.8 mg/m^2^, and infusion time was increased from 30 to 60 min beginning with one patient at dose level 11.7 mg/m^2^. No toxicities ≥grade 3 were reported. MTD was not reached at the highest dose level evaluated. An isolated occurrence of grade 2 increase in alanine aminotransferase/serum aspartate aminotransferase (ALT/AST) in a patient receiving 11.7 mg/m^2^ led the study investigators, in conjunction with the sponsor medical monitor, to enroll three more patients at the 11.7 mg/m^2^ dose to provide assurance of hepatic safety. The patient was a 46-year-old female with infiltrating ductal carcinoma metastatic to liver and bone at baseline. Her ALT and AST increased during 2 weeks of dosing. Serum bilirubin remained within normal limits, and the enzyme levels returned to baseline 2 weeks after dose interruption. The increased ALT/AST did not recur on continued treatment. No other patient in that cohort or in subsequent higher dose cohorts experienced a clinically significant increase in liver enzymes, and this finding did not meet the definition of a DLT. No further DLTs were observed among the three additional patients treated at this dose level.

### Safety

Table 2 lists selected treatment-related AE. Other AE not presented in the table included: infusion site hypersensitivity (*n* = 4 patients, 10.8 %), two patients each with decreased appetite, rhinorrhea and hyperhidrosis (5.4 %), and one patient each with “pain,” candidal infection, hypokalemia, muscle spasm, peripheral neuropathy, tremor, epistaxis, pruritus, macular rash and hot flushes (2.7 %). There were no grade 3 or 4 AE in any patients. All AE were classified as unrelated or unlikely related to EP-100, with the exception of infusion-related dermatologic reactions (IRDRs).

**Table 2:** Selected toxicities (all cycles)

(*N* = 37)	Grade 1/2 No. of patients (%)	Grade 3/4 No. of patients (%)
Constitutional		
Fatigue	4 (10.8)	0
Dermatologic		
Infusion-related dermatologic reactions	10 (27)	0
Hepatic		
AST/ALT	1 (2.7)	0
AP	1 (2.7)	0
Total bilirubin	0	0
Gastrointestinal		
Diarrhea	1 (2.7)	0
Gastroesophageal reflux	1 (2.7)	0
Vomiting	1 (2.7)	0
Hematological		
Hemoglobin	0	0
Platelets	0	0
Neutrophils	0	0

*AST* serum aspartate aminotransferase, *ALT* alanine aminotransferase, *AP* alkaline phosphatase

The most frequent AE, occurring at the time of EP-100 infusions, were IRDRs. IRDRs is a term chosen to describe a spectrum of skin reactions occurring during EP-100 infusion and reported by investigators as “infusion reaction/hypersensitivity,” “cytokine release syndrome,” or “urticaria.” IRDRs were reported in 10 of 37 patients. Symptoms included superficial burning and/or pruritus, with or without diffuse erythema or flushing, and with small papules or atypical hives on the trunk and/or upper extremities. Symptoms were variable in extent and severity, with none more than grade 2. Hemodynamic instability and hypoxia were not observed. Reactions began within 15 min of the drug infusion and began to dissipate almost immediately after cessation of the infusion. Typically, all symptoms disappeared within 30 min and were not associated with major organ dysfunction or clinically significant sequelae. Hematology and chemistry profiles were unchanged, without eosinophilia or indications of inflammatory response. One patient reported accompanying shortness of breath and chest tightness, but had no substernal pain, changes on ECG or in hemodynamic parameters. IRDRs appeared to be patient specific and variable, occurring both with the first and sometimes subsequent infusions. IRDRs did not predictably recur in each patient. Temporary interruption of the infusion allowed the reaction to subside. There was no correlation of IRDRs to tumor type, gender or treatment cycle number (first vs subsequent). No IRDRs were reported in the four lowest dose cohorts (0.6–1.7 mg/m^2^ at dose concentrations of 0.01–0.03 mg/mL). IRDRs incidence was lower when EP-100 infusion times were increased from 30 to 60 min (beginning with cohort 7), which effectively reduced circulating drug concentration by 50 %. IRDRs incidence then increased again in the higher dose cohorts (17.5 mg/m^2^, 0.26 mg/mL).

Management of IRDRs included cessation of the infusion until symptoms resolved. Investigators reported improved symptoms by administering an antihistamine (such as diphenhydramine or hydroxyzine), a corticosteroid (such as dexamethasone) and/or and H2-antagonist (such as famotidine or ranitidine). Once symptoms had resolved and the patient appeared clinically stable, infusions were restarted at a slower rate, without recurrence of symptoms in the majority of patients. After observing repeated IRDRs in one patient at dose level 2.6 mg/m^2^ in Cycle 3, a consistent pre-treatment regimen was recommended for all patients and included an antihistamine (typically hydroxyzine or diphenhydramine), an H2-antagonist such as famotidine or ranitidine and a corticosteroid, such as dexamethasone, to be administered prior to all EP-100 infusions. Despite pre-treatment, IRDRs recurred with subsequent cycles in six patients; in two patients IRDRs occurred twice, and in two patients several occurrences were recorded.

Fourteen SAE were recorded (Table 3). All were grade 1 or 2. All were judged by the investigators as unrelated or unlikely related to EP-100. There was one reported death within 1 month of withdrawal from the trial; the death was attributed to disease progression in a 49-year-old female with serous papillary carcinoma metastatic to liver.

**Table 3:** Treatment-emergent serious adverse events (SAE)

SAE	No. of patients (%)	Dosing, mg/m^2^ (No. of patients)	Relationship to EP-100	Outcome
Gastrointestinal obstruction	2 (5.4)	0.6, 0.9 qw	Unrelated	Resolved with sequelae
Gastrointestinal hemorrhage	1 (2.7)	1.35 qw	Unlikely related	Resolved
Peripheral edema	1 (2.7)	1.35 qw	Unrelated	Resolved with sequelae
Disease progression	1 (2.7)	1.35 qw	Unrelated	Death
Pain	1 (2.7)	1.7 qw	Unrelated	Ongoing
Hydronephrosis	1 (2.7)	11.7 biw	Unrelated	Resolved
Chills	1 (2.7)	11.7 biw	Unrelated	Resolved
Pleural effusion	2 (5.4)	11.7, 26 biw	Unrelated	Resolved
Febrile neutropenia	1 (2.7)	17.5 biw	Unrelated	Resolved
Ascites	1 (2.7)	26 biw	Unlikely related	Resolved with sequelae
Fatigue	1 (2.7)	26 biw	Unrelated	Resolved
Facial paresis	1 (2.7)	40 biw	Unrelated	Ongoing

Qw, weekly; biw, twice weekly

### Pharmacokinetics

**Fig. 2:**
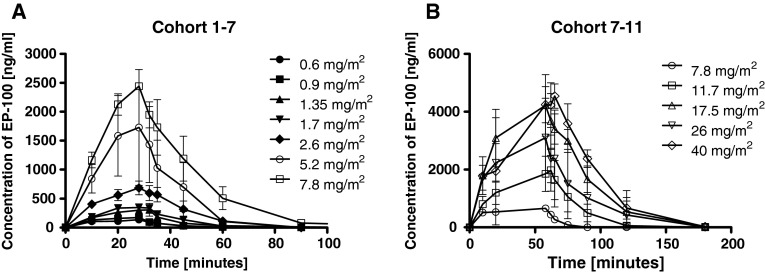
**a** Mean plasma concentration versus time curve by dose level for patients treated with a 30-min infusion of EP-100. **b** Mean plasma concentration versus time curve by dose level for patients treated with a 60-min infusion of EP-100

**Fig. 3:**
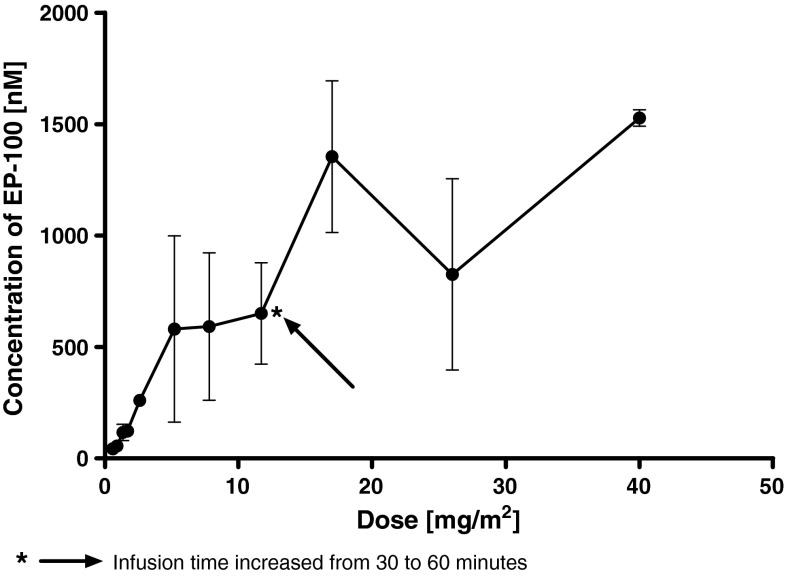
Plasma exposure [plasma concentration (*C*_max_) vs dose level] among 37 patients treated with EP-100 in a first-in-human phase I trial

EP-100 plasma concentrations increased rapidly with intravenous administration, and duration of *C*_max_ levels were extended with lengthening of the infusion time from 30 to 60 min beginning with cohort 7. *C*_max_ was reached within 20–40 min when EP-100 was given over 30 min, and within 40–60 min when EP-100 was given over 60 min in the majority of patients (Fig. 2a, b). EP-100 concentrations declined in an exponential fashion after approximately 40 min in the 30-min infusion cohorts, and after approximately 60 min in the 60-min infusion cohorts. The goodness-of-fit statistic for estimation of *λ*_z_ (*R* ^2^) was >0.8 for EP-100 for all subjects analyzed; hence, *λ*_z_ was considered reliable, and this parameter and associated parameters were reported for all subjects. The increase in *C*_max_ with dose appeared to be slightly greater than dose proportional; however the data were highly variable (Fig. 3). AUC_0–*t*_ and AUC_0–*∞*_ also appeared to increase in a nonlinear manner relative to dose. The percent of AUC extrapolated was less than 20 % for all dose groups, suggesting that the sample collection period was sufficient to characterize the concentration versus time curves. The volume of distribution, *V*_z_, appeared to decrease with an increase in dose. *V*_z_ ranged from 4,346.6 to 1,564.4 mL in going from a dose of 0.6–5.2 mg/m^2^, respectively. Following doses of 7.8 to 17 mg/m2, *V*_z_ remained relatively constant and ranged from ~2,400 to 2,666 mL (~60 mL/kg).

Clearance was variable but generally decreased with an increase in dose and ranged from 299.0 to 142.6 mL/min at a doses of 0.6–5.2 mg/m^2^, respectively. Following doses of 7.8, 11.7 and 17 mg/m^2^, clearance was 223.1, 221.5 and 109.6 mL/min. Clearance was rapid (mean half-life 7.1 ± 3.8 to 15.9 ± 3.6 min). There did not appear to be any correlation of dose with half-life. At doses of 5.2 mg/m^2^ and above, plasma concentrations reached the predicted “therapeutic” concentrations that demonstrated activity in vitro in all pre-clinical studies. At the highest dose level tested, mean *C*_max_ and AUC_0–*∞*_ were 4,760 ± 113 ng/mL and 325,709 ± 73,205 ng*min/mL, respectively.

### Pharmacodynamics

Sustained decreases in LH and FSH in three breast cancer patients were indicative of EP-100’s pharmacological activity on pituitary gonadotropes. One patient treated at dose level of 7.8 mg/m^2^ had an 88 % reduction from baseline FSH (127–15 U/L) and LH reduction of 96 % (24–1 U/L). The second patient (11.7 mg/m^2^) had FSH decrease by 81 % (21–4 U/L) and LH decrease from 1.2 U/L at baseline to less than 1 U/L. The third patient (26 mg/m^2^) had FSH decrease by 85 % (27–4 U/L) and LH decrease by 95 % (27–1.3 U/L).

Antibodies to EP-100 were not detected at any dose level.

### Antitumor activity

Clinical benefit was observed in seven patients (19 %), all of whom had stable disease for 16 weeks or longer while receiving EP-100. Diagnoses of patients with stable disease included carcinoid (0.6 mg/m^2^), pancreatic cancer (0.9 mg/m^2^), two ovarian cancers (2.6 and 11.7 mg/m^2^), two breast cancers (11.7 and 17.5 mg/m^2^) and one endometrial cancer (26 mg/m^2^), with at least 16 weeks’ SD. No objective tumor responses were seen by RECIST version 1.1, and there were no treatment-related decreases in relevant tumor biomarkers (CA-125 or PSA) or CTC values.

## Discussion

EP-100 is a unique, targeted anticancer fusion peptide, designed to be selectively toxic to cancer cells expressing LHRH receptor. The primary objectives of this study were to determine the MTD and DLT of EP-100 and to establish a dose regimen of EP-100 recommended for future phase 2 protocols. MTD was not reached at the highest dose level tested. The recommended dose for phase 2 protocols is 40 mg/m^2^ by IV infusion over 1 h twice weekly for 3 of 4 weeks per cycle at a dose concentration of 0.5 mg/mL. To maximize the chance of therapeutic efficacy, the highest dose level tested was recommended for phase 2 trials.

Results of the study show that EP-100 administered as an IV infusion twice weekly for 3 out of 4 weeks per cycle was well tolerated and displayed reproducible PK and PD profiles. Toxicity was minimal. EP-100 was well tolerated after IV infusion in human subjects with advanced solid tumors. One patient experienced a grade 2 ALT/AST increase at 11.7 mg/m^2^, which reversed after withholding EP-100 for 2 weeks. This finding did not meet criteria for a DLT, and no other DLT were observed among three other patients treated at this or subsequent dose levels. The most frequent AE were IRDRs. AE other than IRDRs were few in number and were classified as unrelated or unlikely to be related to EP-100. All AE, with the exception of IRDRs, are expected and routine for patients with advanced cancers.

The mechanism for EP-100-associated IRDRs is not known. Pre-clinical studies in mice and beagle dogs suggest EP-100 may trigger histamine release based on clinical signs of swelling, redness or raised areas on the face and ears, neck, trunk and/or limbs of affected animals. Clinical signs of histamine release were eliminated in a female dog and lessened in a male dog when diphenhydramine was administered. Therefore, histamine release may cause IRDRs, but it remains unclear how EP-100 triggers IRDRs in humans and why the reaction is seen in skin only. Among patients experiencing IRDRs, hematology and chemistry profiles were unchanged, eosinophilia did not occur and no indicators of inflammatory response were detectable in serum. IRDRs were apparently concentration dependent. No IRDRs were reported in the four lowest dose cohorts (0.6–1.7 mg/m^2^, dose concentration 0.01–0.03 mg/mL). IRDRs incidence was lower when EP-100 infusion times were increased from 30 to 60 min. IRDRs incidence then increased again in the higher dose cohorts above a dose concentration of 0.26 mg/mL, at 17.5 mg/m^2^. Although we observed more IRDRs with increasing dose concentration, IRDRs are less likely dependent on the total administered dose of EP-100. IRDRs were reduced by reducing the infusion rate, thus extending the infusion time, resulting in complete dose administration. Pre-treatment with antihistamines, H2-antagonists and corticosteroids did not fully eliminate IRDRs, but seemed to lessen the severity. None of the patients experienced typical “cytokine-release-type infusion reactions,” suggesting that the cytokine release mechanism is unlikely.

This study also sought to determine the pharmacokinetics of EP-100. During IV infusion, EP-100 concentrations rise rapidly and decrease in an exponential fashion on its completion. EP-100 displays nonlinear pharmacokinetics. The terminal half-life of EP-100 is short, at approximately 15 min at the highest dose tested. There did not appear to be any correlation of dose with half-life. Clearance appeared relatively consistent across dose levels. Inter-subject variability was moderate at each dose level with the %CV of *C*_max_ and AUC_0–*∞*_ values ranging from 2 (at 40 mg/m^2^) to 71.9 (at 5.2 mg/m^2^) and 7.5 (at 1.7 mg/m^2^) to 65 (at 26 mg/m^2^), respectively. The decrease in clearance in the majority of subjects with an increase in dose may be suggestive of saturation of a clearance mechanism for EP-100. The *V*_z_ is consistent with the plasma volume, 40–60 mL/kg, suggesting little extravascular distribution at the higher doses.

Evidence of EP-100’s biological activity is confirmed by sustained decreases in LH and FSH only in three patients (8 %) treated at dose levels 7.6, 11.7 and 26 mg/m^2^. Each of these subjects had breast cancer. This finding suggests the LHRH agonist portion of EP-100 retains the ability to bind and suppress gonadotropin release from the anterior pituitary in some patients. Other pituitary hormones (ACTH and TSH) were not affected, indicating that the reduction in the levels of FSH and LH in plasma of the three patients was mediated via LHRH receptors on gonadotropes. A lack of ADA provides reassurance that prolonged administration of EP-100 does not result in autoimmunity to endogenous LHRH.

EP-100 as a single agent did not produce objective tumor responses. Stable disease for more than 3 months was observed in seven patients (two breast cancers, two ovarian and one each of carcinoid, pancreatic and endometrial cancer). Disease stability recorded in these few patients may have resulted from EP-100, but it could be due to underlying tumor biology. We cannot make a definite conclusion from the available data; further validation of EP-100 clinical activity requires additional clinical trials.

EP-100 was safe even with increasing drug exposure that was achieved by extending the infusion time. Increased infusion time increased circulating drug levels, which rapidly declined after infusion cessation. Further studies for increasing EP-100 exposure could help to optimize dosing and increase antitumor activity. Since EP-100 is comprised of L-amino acids, it is readily degraded by proteases in circulation or released from lysed tumor cells. Improvement of the formulation, for example as a targeted liposome or nanoparticle, could enhance the stability and bioavailability of EP-100.

In vitro studies of EP-100 in combination with P-glycoprotein 1 (pgp)-substrates (doxorubicin, paclitaxel, vinorelbine, vincristine) and non-pgp substrates (cisplatinum, 5-fluorouracil) showed reversion of drug resistance and potentiation of toxicity in LHRH-receptor-positive cell lines [39, 40]. Mechanistic studies suggest that the synergy between EP-100 and drugs such as paclitaxel and doxorubicin were mediated via inhibition of pgp [39, 40]. In an in vivo breast cancer model, combination EP-100 with paclitaxel was well tolerated [Esperance, data on file]. A phase 2 clinical trial testing EP-100 with paclitaxel versus paclitaxel alone for patients with advanced ovarian cancer requiring second- or third-line treatment is ongoing [41].

Pre-clinical and now clinical studies demonstrate that EP-100 is a safe, well-tolerated drug that causes no serious organ toxicity and has reliable pharmacokinetic properties. Dermatologic reactions during EP-100 infusion appear manageable and require no additional premedication to prevent reactions than is given routinely prior to infusions of anticancer agents such as paclitaxel. The PD data support the LHRH agonist activity of the drug. Based on pre-clinical models, further clinical trials of EP-100 in combination with pgp substrates are warranted.

## Electronic supplementary material

Below is the link to the electronic supplementary material.

Supplementary material 1 (DOCX 221 kb)
