# Supplementary material for: Novel LHRH-receptor-targeted cytolytic peptide, EP-100: first-in-human phase I study in patients with advanced LHRH-receptor-expressing solid tumors
Source: Cancer Chemother Pharmacol. 2014 Mar 8;73(5):931–41. doi: 10.1007/s00280-014-2424-x (PMC4000412; doi:10.1007/s00280-014-2424-x)
Supplement: Supplementary file 1 — Supplementary material 1 (DOCX 221 kb) [file 280_2014_2424_MOESM1_ESM.docx]

Supplementary Information

Inclusion Criteria

- Subjects with histologically confirmed solid carcinomas
- Subjects whose tumors express LHRH-receptors in tumor biopsies
- Tumor progression after receiving standard/approved chemotherapy or where there is no approved therapy
- One or more tumors measurable on CT scan or evaluable disease
- Karnofsky performance ≥ 70%
- Life expectancy of at least 3 months
- Age ≥ 18 years
- Signed, written informed consent. Consent must be provided prior to performing any study-related procedures.
- A negative pregnancy test (if female)
- Acceptable liver function:
  - Bilirubin ≤ 1.5 times upper limit of normal (ULN)
  - AST (SGOT), ALT (SGPT) and Alkaline phosphatase ≤ 2.5 times ULN (if liver or bone metastases are present, then ≤ 5 x ULN is allowed)
- Acceptable renal function:
  - Serum creatinine within normal limits, OR calculated creatinine clearance ≥ 50 mL/min/1.73 m2 for subjects with creatinine levels above institutional normal.
- Acceptable hematologic status:
  - ANC ≥ 1500 cells/mm3
  - Platelet count ≥ 100,000 cells/mm3
  - Hemoglobin ≥ 9 g/dL
- Urinalysis:
  - No clinically significant abnormalities
- Acceptable coagulation status:
  - PT ≤ 1.3 x ULN (unless on therapeutic doses of Warfarin)
  - PTT ≤ 1.3 x ULN
- For men and women of child-producing potential, the use of effective contraceptive methods during the study

Exclusion criteria
